# Elusive modes of Foxp3 activity in versatile regulatory T cells

**DOI:** 10.3389/fimmu.2024.1533823

**Published:** 2025-01-15

**Authors:** Minghong He, Yongqiang Feng

**Affiliations:** Department of Immunology, St. Jude Children’s Research Hospital, Memphis, TN, United States

**Keywords:** regulatory T cells, Foxp3, transcription factor, transcriptional cofactor, DNA binding, chromatin

## Abstract

Foxp3-expressing CD4 regulatory T (Treg) cells play a crucial role in suppressing autoimmunity, tolerating food antigens and commensal microbiota, and maintaining tissue integrity. These multifaceted functions are guided by environmental cues through interconnected signaling pathways. Traditionally, Treg fate and function were believed to be statically determined by the forkhead box protein Foxp3 that directly binds to DNA. However, this model has not been rigorously tested in physiological and pathological conditions where Treg cells adapt their function in response to environmental cues, raising questions about the contribution of Foxp3-dependent gene regulation to their versatility. Recent research indicates that Foxp3 primarily functions as a transcriptional cofactor, whose chromatin interaction is influenced by other DNA-binding proteins that respond to cell activation, stimulation, or differentiation. This new perspective offers an opportunity to reevaluate Foxp3’s activity modes in diverse biological contexts. By exploring this paradigm, we aim to unravel the fundamental principles of Treg cell biology.

## Introduction

How suppressor lymphocytes are programmed and how they function are fundamental questions in immunological research. Remarkable progress has been made in understanding immune-suppressive lymphocytes, including Foxp3^+^ and Foxp3^–^ CD8 suppressor T cells, regulatory B cells (Foxp3^–^), Foxp3^+^ CD4 Treg cells, and Foxp3^–^ CD4 regulatory type 1 T (Tr1) cells (1–6). Among these, Foxp3-expressing CD4 Treg cells have garnered significant attention due to their well-defined lineage and crucial functions, including the suppression of autoimmune responses and antitumor immune responses (2). Foxp3 is widely recognized as the lineage determinant of CD4 Treg cells and is not expressed meaningfully by non-T cell types. It is also transiently upregulated in human conventional T (Tcon) cells upon activation, or in mouse Tcon cells likely during abortive Treg development (7–9). In this review, we delve into the transcriptional mechanisms that govern the diverse biological functions of mouse CD4 Treg cells.

**Figure 1:**
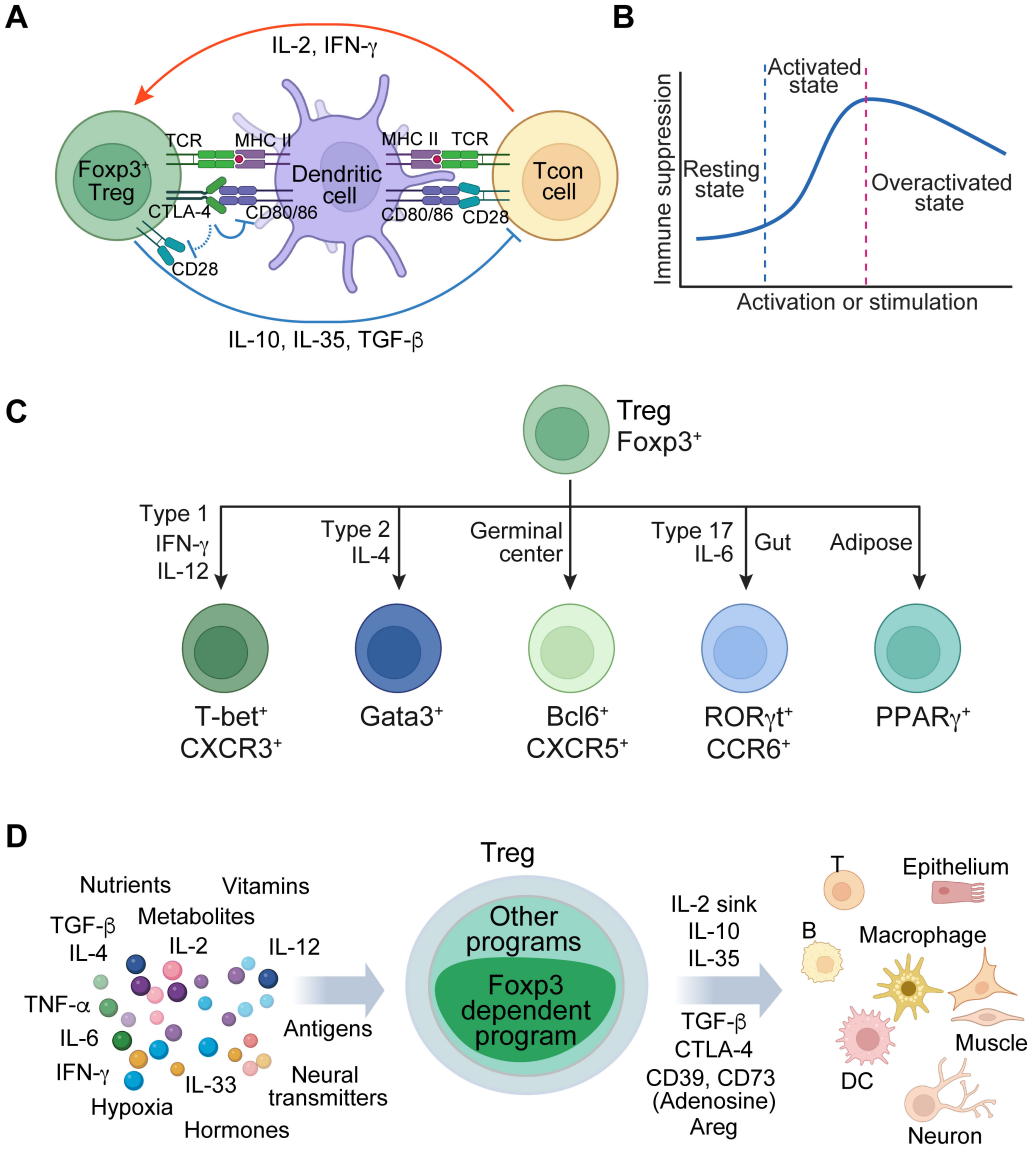
Foxp3 regulates Treg cell fate and functions across different contexts. **(A)** A simplified model of Treg immune suppressive function involving the interactions among Treg cells, antigen-presenting cells, and conventional T (Tcon) cells. Treg cells suppress antigen presentation and co-stimulation from dendritic cells and inhibit Tcon cells through various mechanisms, including acting as an IL-2 sink and secreting immune suppressive factors like IL-10, IL-35, and TGF-β. Meanwhile, dendritic cells and Tcon cells enhance Treg cell function via antigen presentation and cytokines such as IL-2 and IFN-γ. **(B)** A schematic illustrates the regulatory mechanisms governing the flexibility of Treg cell function in response to external stimuli. In the resting state, Treg cells exhibit diminished function. Upon activation through antigen and/or cytokine stimulation, they augment their immune regulatory capabilities. However, excessive activation, particularly in severe inflammatory conditions, can lead to the downregulation of Treg function. **(C)** Treg cells acquire specialized functions through differentiation or adaptation in specific tissue environments, which are characterized by the expression of transcription factors, such as T-bet, Gata3, Bcl6, RORγt, and PPARγ. **(D)** A schematic illustrates the multifaceted regulation of Treg cells by diverse tissue environmental cues, such as antigens, cytokines, nutrients, and metabolites. This regulation facilitates the control of a broad spectrum of immune and non-immune cells involved in diverse biological processes, including tolerance to self-antigens and foreign antigens, anti-viral and anti-tumor responses, tissue repair and regeneration, and metabolic regulation.

CD4 Treg cells, induced in the thymus or periphery, play a crucial role in suppressing immune responses and ensuring tolerance to non-harmful self and foreign antigens. They achieve this through various mechanisms, including inhibiting co-stimulatory signals via CD80/CD86, competing for IL-2 (IL-2 sink), and secreting inhibitory cytokines such as IL-10, IL-35, and TGF-β (**Figure 1A**) (2, 10). They are dynamically regulated by their activation status and environmental cues to precisely adjust their regulatory activity. For instance, upon activation by antigens and cytokines, Treg cells exhibit heightened immune suppression, whereas excessive stimulation in inflammatory conditions may lead to a downregulation of their function (11–13) (**Figure 1B**). They differentiate into subtypes to acquire specialized immune suppression capabilities upon exposure to distinct cytokines or specific tissue environments. This is characterized by the expression of unique transcription factors, including but not limited to T-box transcription factor T-bet (encoded by *Tbx21*) in type one inflammatory conditions such as during antitumor immune responses, GATA binding protein 3 (Gata3) in type two conditions, B-cell lymphoma 6 (Bcl6) in germinal centers, retinoid orphan receptor gamma t (RORγt) under type 17 conditions at mucosal interfaces exposed to food and microbiota, and peroxisome proliferator-activated receptor gamma (PPARγ) in adipose tissue (14–19) (**Figure 1C**). They can also develop innate immune functions in response to tissue damage, as illustrated by the stimulation of alarmin IL-33 (20, 21). Apart from their immune regulatory activity, Treg cells are empowered to maintain tissue integrity and promote repair and regeneration (20, 22, 23). These examples demonstrate the versatile biological functions of Treg cells.

Treg cell fate and function are predominantly regulated by Foxp3. This raises questions regarding the mechanisms by which Foxp3-dependent gene regulation controls versatile Treg cells in response to diverse environmental cues, including antigen stimulation, cytokines, metabolites, nutrients, hypoxia, hormones, neurotransmitters, and various target cell types (**Figure 1D**). The notion that Foxp3 functions as a transcription factor has been pivotal in Treg cell biology, implying that it confers cell fate and immune regulatory function in a static manner by directly binding to target DNA sequences to regulate gene expression. Nevertheless, this model remains uncertain due to the absence of suitable methods for directly testing it in physiological settings. Our recent reassessment of Foxp3 activity in native conditions has revealed that Foxp3 interacts with chromatin through other DNA-binding proteins that are induced by cell activation, stimulation, or differentiation (defined as immunological contexts) (24). In this instance, Foxp3 functions more like a transcriptional cofactor. Here, we delve into this novel paradigm and investigate the elusive activity modes of Foxp3 that underlie the versatile functions of Treg cells.

## Dynamic Foxp3-chromatin interaction in various biological contexts

**Figure 2:**
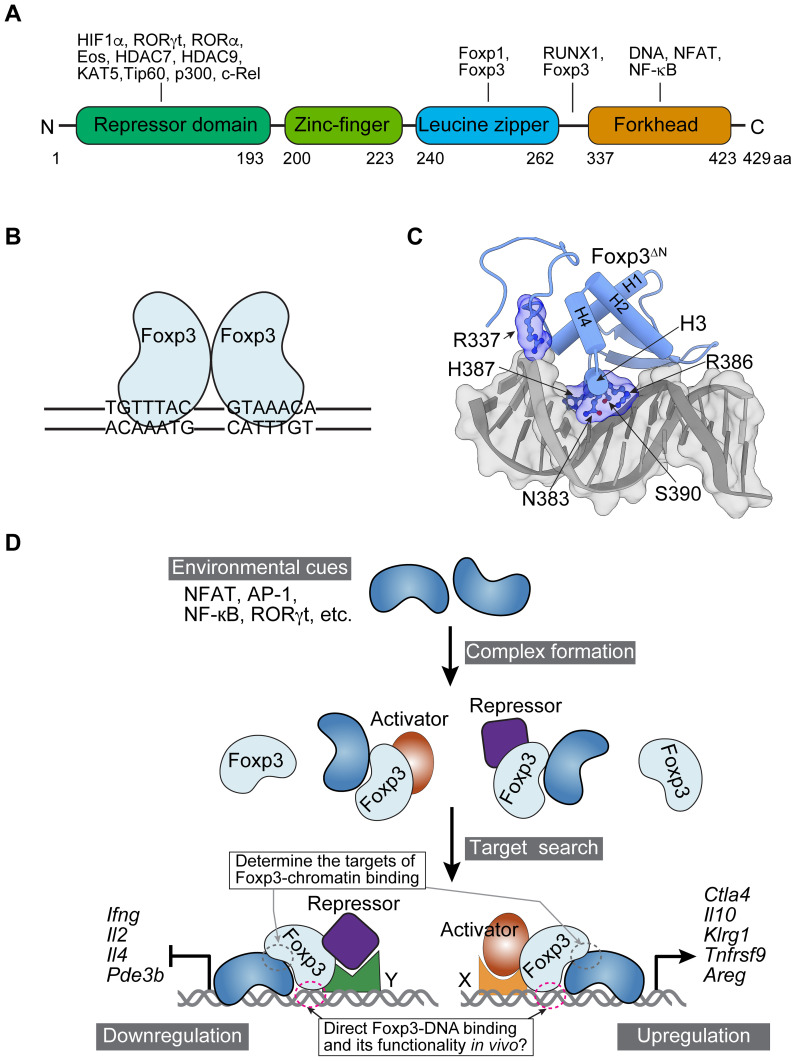
Foxp3 protein domains, interacting proteins, 3D structure, and dynamic interactions with chromatin. **(A)** The domains and representative interacting proteins of murine Foxp3. Note that the domains are not drawn to scale. Human Foxp3 is highly conserved in amino acid sequence and domain structure (not shown). **(B)** A schematic illustrates Foxp3 binding as a dimer to DNA with two inverted FKHM sequences (TGTTTAC), based on *in vitro* binding results. **(C)** A 3D structure of Foxp3 FHD and a DNA probe (PDB: 7TDX; prepared with ChimeraX-1.8, https://www.rbvi.ucsf.edu/chimerax/). Amino acid residues that directly interact with DNA are labeled. Foxp3^ΔN^ refers to recombinant Foxp3 lacking N-terminal domains; H1-H4 denote α-helices. **(D)** A model illustrates the dynamic regulation of Foxp3-chromatin interaction. Environmental cues refer to the conditions Treg cells are exposed to in particular tissues, including antigens, cytokines, hormones, nutrients, metabolites, and neurotransmitters. They induce DNA-binding proteins such as NFAT, AP-1, NF-kB, and RORγt in Treg cells to form complexes with Foxp3. This recruits Foxp3 to chromatin or stabilizes Foxp3-chromatin interaction. Transcriptional activators or repressors associate with Foxp3 to upregulate (right) or downregulate (left) gene expression through sequence-specific readers X and Y. Examples of upregulated and downregulated genes are illustrated. As a result, Foxp3 modulates Treg cell function according to tissue environmental cues. For simplicity, other regulations, such as epigenetic modifications, are not depicted in the model. Consequently, Foxp3 regulates the target genes of NFAT, AP-1, NF-κB, RORγt, and other transcription factors, contrasting with the previous assumption that suggested Foxp3 binds to DNA directly and recruits these factors to modulate its target gene expression. Foxp3 also regulates target genes in trans or indirectly, and its chromatin binding may be influenced by epigenetic modifications, such as histone acetylation and DNA methylation, as well as chromatin accessibility, adding further layers of regulation (not shown).

In the simplified static model of Treg fate and function determination, Foxp3 is commonly viewed as a transcription factor that directly binds to DNA through its forkhead domain (FHD). This binding action enables Foxp3 to either upregulate or downregulate the expression of target genes by associating with transcriptional activators (such as RelA, Ikzf2, and Kat5) or repressors (such as Ezh2, Ikzf1, Ikzf3, and YY1) (25–28) (**Figure 2A**). This assumption mainly arises from the facts that FOX family proteins, including Foxp3, possess FHDs, and that *in vitro* binding assays and 3D structures of purified Foxp3 FHD demonstrate strong binding to DNA probes with canonical forkhead motifs (FKHM) and T_n_G repeats (29–32) (**Figures 2B, C**). Chromatin immunoprecipitation (ChIP) based on chemical cross-linking indicates Foxp3 binding to genes differentially expressed in Treg and Tcon cells (33–35). These observations support the presumed role of Foxp3 as a transcription factor, but they do not rule out the possibility of it acting as a transcriptional cofactor if its FHD is not utilized for DNA binding.

We recently examined Foxp3-chromatin interactions in several representative physiological and pathological settings using CUT&RUN sequencing, which probes protein-chromatin binding without crosslinking (36), leading to unexpected observations (24). First, Foxp3 exhibits distinct binding patterns during Treg cell activation or adaptation to the tumor environment *in vivo*. Notably, the upregulation of genes such as *Il10*, *Ctla4*, and *Klrg1* is correlated with increased Foxp3-chromatin binding and enhanced suppressive function of Treg cells. These regions are enriched with transcription factor motifs, including AP-1, NFAT, and NF-κB. Second, acute stimulation by TCR agonists or recombinant IL-2 rapidly modifies Foxp3-chromatin binding at specific targets. Conversely, cyclosporin A blockade of calcineurin activity *in vivo* downregulates NFAT signaling and significantly impairs Foxp3 binding. Thus, TCR or IL-2R signaling is required and/or sufficient for enhanced Foxp3-chromatin binding at many targets. Third, Foxp3 forms complexes with Ets1 and AP-1 proteins, such as Batf that is highly expressed in tumor-infiltrating Treg cells (37). CRISPR deletion of *Ets1* or *Batf* reduces Foxp3-chromatin binding, while Batf overexpression enhances it. Therefore, Foxp3 appears to interact with chromatin in a context-dependent manner, enabling Treg cells to perform a wide range of functions, which contradicts the previously assumed static model (**Figure 2D**). Subsequently, transcriptional repressors such as Ikzf1, NuRD, and Ezh2, along with activators like p300, RelA, and NFAT1, and other regulators recruited by Foxp3 (if they do not determine Foxp3-chromatin binding in the first place) in a locus-specific manner using DNA sequence readers, will either repress or promote gene expression (25–28, 38). Factors such as TCF1 may regulate gene expression in Treg cells independently of direct interaction with Foxp3 (39, 40).

This model is consistent with previous observations that transcription factor RORγt, induced in Treg cells by food antigens or microbiome (41), binds and translocates Foxp3 into the nucleus even if Foxp3’s nuclear localization signal at FHD is deleted (42). RORγt’s association may also recruit Foxp3 to chromatin, modulating RORγt’s target gene expression, such as inhibiting IL-17 and IL-23.

Based on these representative settings, an emerging model proposes that Foxp3 regulates gene expression through dynamic interactions with chromatin in response to cellular activation, stimulation, or differentiation. This model suggests that Treg fate and function are determined by context-dependent Foxp3-chromatin binding, which is influenced by associated transcription factors. Given the diverse immunological contexts, including diseases, it is imperative to thoroughly validate this model and elucidate the precise regulation of the interaction between Foxp3 and chromatin by various tissue environmental cues. This should involve identifying target genes that exhibit differential expression and Foxp3 binding, utilizing advanced transcriptomics and ChIP technologies such as CUT&RUN or CUT&Tag sequencing in native physiological and pathological conditions (36, 43). Further research is necessary to elucidate the causal role of dynamic Foxp3-chromatin binding in determining Treg cell function. This can be accomplished through reporter and Treg functional assays, conducted on a case-by-case basis or for specific gene subsets, after selectively disrupting or introducing Foxp3-chromatin binding.

Dynamic regulation of Foxp3-chromatin binding raises several pertinent questions: How is Foxp3’s target specificity determined? Does the FHD of Foxp3 bind to DNA *in vivo*, and if not, what is its actual function? Is Foxp3 in excess in the steady state with fewer immunological cues, such that partial loss of Foxp3 would not impair Treg cell function? Could Foxp3 be outnumbered by inflammation-driven DNA-binding proteins upon intense stimulation, such as strong antigen engagement or severe inflammation, thereby downregulating Treg cell function? In this context, can the overexpression of wild-type Foxp3 or the provision of engineered Foxp3 alleles overcome this limitation and more effectively suppress inflammatory diseases?

In addition to genes directly controlled by Foxp3 through chromatin binding, Foxp3 can also regulate gene expression in trans or indirectly (44). Foxp3-chromatin binding may be affected by chromatin and epigenetic modifications, such as accessibility, histone modifications, and DNA methylation. These factors could be context-dependent, varying with genomic loci and biological conditions, and introduce additional regulatory layers that require comprehensive exploration. For clarity, we focus on Foxp3 chromatin interaction in cis alongside associated DNA-binding proteins, though the concept is applicable to more complex scenarios.

## Paradox in determining target-specific DNA binding of Foxp3 complexes

Foxp3, apart from its intricate transcriptional regulation of expression (45–47), forms complexes with over 300 proteins, including transcription factors, transcriptional cofactors, chromatin remodelers, post-translational modifying enzymes, and RNA-binding proteins (25, 48). Given that Foxp3 and associated transcription factors like NFAT, RORγt, AP-1, and NF-κB can directly bind to DNA *in vitro*, a question arises regarding how the target specificity of Foxp3-chromatin binding is determined. In one scenario, it is possible that Foxp3 completely determines target specificity by binding to DNA through its FHD, assuming Foxp3 functions as a transcription factor. In this instance, Foxp3 might preferentially bind to DNA sequence motifs that have been identified through *in vitro* Foxp3-binding assays, such as FKHM. In another scenario, Foxp3-associated transcription factors primarily determine target specificity, leading to the absence of enrichment of FKHM or T_n_G repeats at Foxp3 peaks. This possibility aligns with most reported Foxp3-binding sites (24, 35, 44). Foxp3’s FHD might be hindered by associated proteins that bind to DNA, repurpose for other functions, or bind too weakly or transiently for current detection methods. Consequently, Foxp3 behaves more like a transcriptional cofactor. In this instance, could direct Foxp3-DNA binding, if it occurs, influence target specificity or binding affinity? A comparison of Batf-binding sites in activated Treg cells, T helper 2 (Th2) cells, and T helper 17 (Th17) cells revealed only a few Treg-specific Batf peaks (24, 49, 50). This finding implies that Foxp3 exerts a negligible influence on Batf binding.

Alternatively, both Foxp3 and its associated transcription factors may contribute to target-specific binding, albeit with varying degrees of influence that require experimental verification. This aligns with the observation that Foxp3 facilitates Foxp3-NFAT or Foxp3-Runx1 binding to DNA substrates containing inverted FKHM adjacent to NFAT or RUNX motifs (31). Given the diverse Foxp3 complexes and the Foxp3-dependent gene regulation, it is plausible that all these activity modes may exist, each with varying preferences for targets or biological settings.

The model of Foxp3 as a transcriptional cofactor is supported by observations that point mutations at the α-helix directly interacting with DNA (H3 in **Figure 2C**) disrupt Foxp3-DNA binding *in vitro* but do not affect chromatin binding and *Il2ra* and *Ctla4* expression, as long as protein levels are similar to wild-type Foxp3 (24). Foxp3 mutants A372P and combined W348Q M370T A372P prevent Foxp3 dimerization and DNA binding *in vitro*, yet result in few changes in Foxp3-chromatin binding (29–32, 51). Foxp3-F324L, situated between the leucine zipper and FHD, significantly impairs DNA binding *in vitro*, presumably by affecting domain-swapped dimerization (31, 52); however, knock-in mice remain healthy (52).

Therefore, in the dynamic Foxp3-chromatin interaction model, Foxp3 binding at most genomic targets is determined by other DNA-binding proteins associated with Foxp3 (**Figure 2D**). This elucidates how certain Foxp3 mutants influence gene regulation and disrupt Treg cell function. For example, six alanine replacements from amino acid 176 and A384T may alter chromatin binding through associated proteins to perturb gene expression, rather than altering target interaction through impaired Foxp3-DNA binding per se (25, 53). Furthermore, alterations in Foxp3-interacting proteins, which impact both chromatin binding and regulatory function, could also be a consequence of immune dysregulation in animals with these mutations, resulting in heightened TCR and cytokine stimulations that lead to higher nuclear localization of NF-κB, NFAT, STAT, and AP-1 proteins, etc. Separating the primary and secondary effects is crucial for comprehending the nature of this dysregulation, which was absent in previous studies.

Crucially, dynamic Foxp3-chromatin interaction implies a distinct mechanism of Foxp3-dependent gene regulation underlying Treg fate and function. In this model, the expression of target genes for NFAT, NF-κB, AP-1, RORγt, and other transcription factors is modulated through attached Foxp3 and its complex components (**Figure 2D**). This contrasts with the previous assumption that Foxp3 binds to presumably novel DNA targets and recruits these transcription factors and other proteins to control its target genes’ expression (25, 44, 48, 54). Clarification of the regulatory hierarchy of Foxp3 offers novel insights into the principles governing the fate and function of Treg cells, a cornerstone of Treg biology. This has long been perplexing because Foxp3 occupies a chromatin landscape established in precursor cells, and Foxp3 target genes are also regulated by other transcription factors present in Tcon cells (35, 54, 55). The dynamic Foxp3-chromatin interaction model presents a paradigm for extensive exploration. It suggests that Foxp3’s modulation of preexisting gene regulatory circuits ultimately determines Treg fate and function.

Direct Foxp3-DNA binding might be pertinent to specific contexts where Foxp3 can directly bind to its own DNA targets to regulate gene expression, as per the conventional model. These genes would be enriched with FKHM or T_n_G repeats, as evidenced by *in vitro* DNA-binding assays. However, the genes in this category require experimental verification with increased sensitivity to detect direct Foxp3-DNA interaction. It would be crucial to determine the significance of this mechanism and identify the immunological contexts involved, such as specific tissue environments or activation status.

Overall, Foxp3 appears to predominantly function as a transcriptional cofactor, with its chromatin binding determined by the associated DNA-binding factors.

## Intricate functions of Foxp3’s forkhead domain

The dynamic interaction between Foxp3 and chromatin indicates that the function of the Foxp3 FHD requires reassessment in physiological and pathological settings. Foxp3 mutations affecting *in vitro* DNA binding provide valuable clues. For example, mutations of the α-helix H3 that eliminate DNA binding *in vitro* destabilize Foxp3 *in vivo* when expressed in Tcon cells (24). Foxp3 mutation R337Q reduces both DNA-binding affinity *in vitro* and protein stability *in vivo* (31, 52). Therefore, the direct binding of Foxp3 to DNA appears to have been repurposed to stabilize the Foxp3-chromatin complex. The correlation between DNA binding and Foxp3 stability raises a question about the underlying mechanisms. Paradoxically, if Foxp3 primarily functions as a transcriptional cofactor in most cases, its DNA-binding activity may contribute little to determining target specificity. Foxp3-DNA binding *in vivo* may not be as strong as *in vitro* binding assays indicate, contributing to Foxp3 protein stability but not target-specific chromatin binding. It is also possible that the Foxp3 FHD has been repurposed for other functions besides DNA binding. Therefore, measuring direct Foxp3-DNA interactions in their natural environment and evaluating its significance are crucial steps in resolving this paradox.

In addition to contributing to DNA binding and protein stability, Foxp3’s FHD unexpectedly interacts with various protein partners, including Runx1, NFAT, and Stk4-NF-kB (30–32, 56). If both Foxp3’s FHD and its associated transcription factors bind to DNA, regardless of binding affinity or target specificity determination, they may loop genetic elements from a distance or alter the local DNA conformation, thereby playing distinct roles in Foxp3-dependent gene regulation (51, 57, 58). These interactions indicate that Foxp3’s FHD may have diverse functions. Are all of these roles essential for Foxp3’s overall function, or are they specific to certain target genes or contexts?

Finally, Foxp3 protein stability has been associated with post-translational modifications, including acetylation and ubiquitination, as well as proteasome-mediated degradation (59–61). The precise mechanism by which FHD mutations affecting Foxp3-DNA binding *in vitro* induce Foxp3 degradation through ubiquitination and proteasome-dependent pathways remains uncertain. It is also crucial to investigate whether this feature modulates the magnitude or duration of Foxp3-dependent gene regulation and Treg function in response to specific environmental cues, particularly severe inflammation.

## Concluding remarks

Despite over two decades of intensive research, the precise mechanistic role of the Foxp3 protein in controlling Treg fate and function remains elusive. Recent studies have provided valuable insights into this complex process. By integrating the available data, we propose a model suggesting that Foxp3 primarily functions as a transcriptional cofactor in most cases, with its DNA-binding domain adapted for other functions. This refined view of Foxp3’s biochemical activity provides a new perspective to reassess its protein structure and function in diverse physiological and pathological conditions. This new perspective offers insights into the nature of Treg fate and the versatile functions determined by Foxp3-dependent gene regulation. It may also apply to human CD4 Foxp3^+^ Treg cells, given the remarkable conservation of Foxp3 protein sequence and structural domains as well as Treg cell function. The novel paradigm derived from this study will facilitate more effective and precise engineering of the Foxp3 protein, which has eluded successful attempts. This would enhance the functionality of Treg cells for the treatment of associated diseases.

Similarly to Foxp3, other transcription factors traditionally regarded as direct DNA-binding proteins may also function as transcriptional cofactors *in vivo*, necessitating extensive investigation. Furthermore, given that Foxp3 is not indispensable for microbiota-induced CD4 Treg cells under certain conditions in the gut, Tr1 cells, regulatory B cells, and some suppressor CD8 T cells (1, 4, 5, 62), understanding the regulatory principles at the transcriptional level would provide important insights into the programming of T-cell immune suppressive function.

## Data Availability

The raw data supporting the conclusions of this article will be made available by the authors, without undue reservation.
